# Progression and Differentiation of Alveolar Rhabdomyosarcoma Is Regulated by PAX7 Transcription Factor—Significance of Tumor Subclones

**DOI:** 10.3390/cells10081870

**Published:** 2021-07-23

**Authors:** Klaudia Skrzypek, Grażyna Adamek, Marta Kot, Bogna Badyra, Marcin Majka

**Affiliations:** Department of Transplantation, Faculty of Medicine, Institute of Pediatrics, Jagiellonian University Medical College, 30-663 Krakow, Poland; grazynadamek@gmail.com (G.A.); marta.kot@uj.edu.pl (M.K.); bogna.badyra@gmail.com (B.B.)

**Keywords:** PAX7, transcription factors, microRNA, surface markers, differentiation, tumor progression, rhabdomyosarcoma, cell subclones, cell lines instability

## Abstract

Rhabdomyosarcoma (RMS), is the most frequent soft tissue tumor in children that originates from disturbances in differentiation process. Mechanisms leading to the development of RMS are still poorly understood. Therefore, by analysis of two RMS RH30 cell line subclones, one subclone PAX7 negative, while the second one PAX7 positive, and comparison with other RMS cell lines we aimed at identifying new mechanisms crucial for RMS progression. RH30 subclones were characterized by the same STR profile, but different morphology, rate of proliferation, migration activity and chemotactic abilities in vitro, as well as differences in tumor morphology and growth in vivo. Our analysis indicated a different level of expression of adhesion molecules (e.g., from VLA and ICAM families), myogenic microRNAs, such as miR-206 and transcription factors, such as MYOD, MYOG, SIX1, and ID. Silencing of PAX7 transcription factor with siRNA confirmed the crucial role of PAX7 transcription factor in proliferation, differentiation and migration of RMS cells. To conclude, our results suggest that tumor cell lines with the same STR profile can produce subclones that differ in many features and indicate crucial roles of PAX7 and ID proteins in the development of RMS.

## 1. Introduction

Myogenesis and myogenic differentiation are processes of muscle formation that are regulated by a number of transcription factors [1]. Myogenesis involves signaling pathways consisting of myogenic regulatory factors (MRF, such as MYF5, MYOD, MYOG, and MRF4), paired-homeobox transcription factors (PAX3 and PAX7) and sine oculis–related homeobox (SIX1 and SIX4) [2]. Early lineage specification is regulated mainly by SIX1/4 and PAX3/7 transcription factors, whereas MYF5 and MYOD commit cells to the myogenic program. Subsequently, myogenin (MYOG) and MRF4 expression is required for the fusion of myocytes and the formation of myotubes [2,3,4,5]. Interestingly, cells in which PAX7 expression remains unchanged recreate a pool of undifferentiated satellite cells [2].

Another family of factors involved in the intramuscular differentiation are inhibitors of DNA binding/differentiation (IDs), belonging to the family of bHLH type transcription factors. ID1-4 proteins are inhibitors of DNA binding and inhibitors of differentiation. They keep cells in an undifferentiated state and allow the preservation of the features typical of stem cells, therefore their production occurs mainly during embryogenesis and in stem cells, while their level decreases and is mostly undetectable in differentiated cells of the mature organism [6]. The ID1 and ID2 proteins were shown to interact strongly with MYOD and MYF5 and weakly with myogenin and MRF4, while ID3 interacts strongly with all MRFs [7].

Myogenic factors may regulate normal myogenesis and regenerative processes, but they may also play a role in pathologic myogenic differentiation. Incorrect signaling pathways may lead to the development of rhabdomyosarcoma (RMS) [1], which is the most frequent soft tissue tumor in children. Analysis of the transcriptomes of abnormally developed muscle cells and RMS cells shows significant similarities between them [8]. Accordingly, RMS cells are characterized by altered levels of transcription factors regulating the myogenesis process [9]. Several RMS subtypes are distinguished, including the embryonic subtype (ERMS), which accounts for approximately 70% of all RMS cases, and the alveolar (ARMS) subtype which is usually associated with poor prognosis. Important factors regulating RMS progression include surface receptors, such as the CXCR4 [10] and c-MET [11,12,13], SNAIL transcription factor [14,15,16], and microRNAs that may post transcriptionally regulate expression of many genes [17,18,19]. Moreover, in 80% of ARMS there are two characteristic translocations: PAX3-FOXO1 and PAX7-FOXO1, which account for approximately 75% and 25% of all translocations appearing in RMS, respectively [1]. Nevertheless, the role of PAX7 in ARMS negative for PAX7-FOXO1 fusion has so far been described to a limited extent in the literature. In particular, it has been determined that miR-206 mediated downregulation of PAX7 expression is essential for the progression of ARMS cell differentiation [20].

A useful tool allowing to expand knowledge in cancer research are in vitro cell cul-tures, which can simulate in vivo tumor evolution. Both during the culture of the cell line in vitro and tumor growth in vivo, clonal selection of cells can take place, which leads to the isolation of a population with evolutionarily more favorable features, such as better survival, faster growth or the ability to colonize other anatomical sites of the body in the case of growth in vivo [21,22]. Therefore, the features of neoplastic cells—such as proliferative, metabolic, chemotactic, or migratory activity—influence the malignancy of the tumor, its metastatic abilities, and the related effectiveness of therapy and prognostic favorability [23]. There are studies in which genetic instability of lines during culture was treated as a model of clonal evolution of tumors [24,25] and as a tool enabling the identification of molecular factors influencing tumor progression [26,27].

Therefore, in our current studies we aimed to perform a comparative analysis of the cells of two subclones of the ARMS RH30 cell line, PAX7 negative (PAX7^−^) and PAX7 positive (PAX7^+^), to identify novel molecular factors influencing tumor progression, based on the differences between these subclones and in comparison to other RMS cell lines. Furthermore, the role of PAX7 was validated in experiments with siRNA.

## 2. Materials and Methods

### 2.1. Cell Culture

RMS cell lines—RH30, RH41, and RD—were kindly provided by Dr. PJ Houghton (Center for Childhood Cancer, Columbus, OH, USA), additionally RH30 cell line was ordered from ATCC (American Type Culture Collection, Manassas, VA, USA), and RH18 cell line from DSMZ (Leibniz Institute DSMZ-German Collection of Microorganisms and Cell Cultures in Germany). The cells were cultured in DMEM high-glucose medium (PAA Laboratories GmbH, Pasching, Austria/Lonza Group Ltd., Basel, Switzerland) supplemented with FBS (EURx, Gdansk, Poland) and 50 μg/mL gentamicin (Lonza) at 37 °C, 5% CO_2_ and 95% humidity. The cell lines were routinely tested for *Mycoplasma spp*. contamination using MycoAlert™ Mycoplasma Detection Kit (Lonza). RMS cell line authentication was performed by STR profiling using AmpFlSTR SGM PLUS Kit (Applied Biosystems, Foster City, CA, USA) and sequencing apparatus ABI Prism 310 Genetic Analyser (Applied Biosystems) according to the manufacturer’s protocol. The results were compared with literature data described in the Expasy Cellosaurus database [28].

The RMS cell lines were differentiated in DMEM low-glucose medium (Lonza or PAA) supplemented with 2% horse serum (HS) (Gibco, BRL Grand Island, NY, USA), as described previously [15], or with the same medium with 5 μM ATRA, as described previously [29,30,31], for 6–7 days. Those two experimental protocols of differentiation in vitro were compared in their effectiveness of myogenic factors induction in RH30 subclones.

The cellular morphology was visualized using Wright’s stain (Sigma-Aldrich, St. Louis, MO, USA).

### 2.2. Transfection with siRNA

RH30 PAX7^+^ cells were transfected with 20 nM siRNA against PAX7 (combination of two Silencer Select siRNA ID variants: s10070 and s10071, Ambion Inc., Austin, TX, USA) or scrambled control siRNA (Silencer Select Negative Control #1 siRNA, cat. 4390844, Ambion) using Lipofectamine RNAiMAX (Invitrogen) transfection reagent according to vendor’s protocol, as described previously [14]. Expression levels, proliferation and migration were estimated within several days after transfection.

### 2.3. Proliferation, Cell Cycle, and BrdU Incorporation

RH30 cells were seeded on 24-well plates. After 24 h, medium was changed and 24, 48, and 72 h later cells were counted in a Bürker hemocytometer chamber to evaluate the proliferation rate in the standard growth medium.

For the assessment of DNA content and BrdU incorporation, RH30 cells were seeded at density 200,000 cells per one well of 6-well plate and cultured in three different conditions: (1) standard culture medium DMEM HG (high-glucose) with 10% FBS for three days, (2) starvation medium DMEM with 0.5% BSA for 48 h and then in DMEM with 10% FBS for subsequent 24 h, and (3) in differentiating medium DMEM low-glucose (LG) with 2% HS for three days. Subsequently, the cells were analyzed using APC BrdU flow Kit (BD Pharmingen, CA, USA) using Attune flow cytometer (ThermoFisher Scientific), according to vendor’s protocol.

### 2.4. Scratch Assay

Confluent RH30 cells were treated with DMEM HG medium with 0.5% BSA for 24 h to inhibit proliferation of the cells. Subsequently, a scratch was generated with a pipette tip. Photographs were taken after 24 h and they were analyzed using ImageJ software (National Institutes of Health, Bethesda, MD, USA), as described previously [14].

### 2.5. Chemotaxis Assay

Chemotaxis of RH30 cells to 10% FBS, 20 ng/mL HGF (R&D System) and 100 ng/mL SDF-1 (Peprotech, Rocky Hill, NJ, USA) was evaluated using modified Boyden’s chamber with 8 μm pore polycarbonate membrane inserts (Transwell; Corning Life Sciences—PZ HTL SA, Warsaw, Poland), as described previously [13]. 0.5% BSA served as a negative control.

### 2.6. Flow Cytometry

For evaluation of CXCR4 receptor expression levels, RH30 cells were labeled with PE-conjugated anti-human CXCR4 antibody (Becton Dickinson, Franklin Lakes, NJ, USA) or mouse IgG1 isotype control (Becton Dickinson) conjugated with PE respectively. The expression level of other surface markers was evaluated using Lyoplate technology (Lyoplate Screening Panel, Becton Dickinson) according to the manufacturer’s protocol. The stained cells were acquired by the usage of Attune Next Flow Cytometer and analyzed using Attune NxT Software v2.2 (Thermo Fisher Scientific, Waltham, MA, USA).

### 2.7. RNA Isolation and Reverse Transcription

Total RNA was extracted using the GeneMATRIX Universal RNA/miRNA Purification Kit (EURx), according to the vendor’s protocol. Reverse transcription of mRNA was performed using MMLV reverse transcriptase (Promega, Madison, WI, USA) according to the manufacturer’s protocol. Reverse transcription miRNA was performed using the Universal cDNA Synthesis Kit (Exiqon, Vedbaek, Denmark) or miRCURY LNA RT Kit (Qiagen, Hilden, Germany), according to the manufacturer’s protocol.

### 2.8. Quantitative Real-Time PCR

Gene expression was determined by qRT-PCR analysis using Quant Studio 7 Flex System (both from Applied Biosystems, Foster City, CA, USA), Blank qPCR Master Mix (EURx) and the indicated Taq-Man probes (Applied Biosystems): human: GAPDH (Hs02758991_g1), MYF5 (Hs00271574_m1), MYOD (Hs00159528_m1), MRF4 (Hs00242962_m1) PAX7 (Hs01547104_g1), PAX3 (Hs00240950_m1), SIX1 (Hs00195590_m1), SIX4 (Hs00213614_m1), MEF2A (Hs01050409_m1), MSTN (Hs00976237_m1), MYOG (Hs01072232 m1), CXCR4 (Hs00237052_m1), ID1 (Hs03676575_s1), ID2 (Hs04187239_m1), ID3 (Hs00954037_g1), ID4 (Hs02912975_g1). The mRNA expression level for all of the samples was calculated using normalization to the housekeeping gene GAPDH (Hs99999905_m1), using the 2^−ΔCt^ method.

PAX3-FOXO1 level was measured using SYBR Green qPCR Master Mix (EURx) and the following primers:

PAX3-FOXO1 forward: 5′-AACCCCACCATTGGCAATG-3′
PAX3-FOXO1 reverse: 5′-ACCCTCTGGATTGAGCATCCA-3′

For the evaluation of miRNA expression by quantitative real-time PCR, SYBR Green qPCR Master Mix (EURx) with LNA™ PCR primer set (Exiqon) or miRCURY LNA miRNA PCR Assay (Qiagen) for human miR-1-3p, miR-133a-3p, miR-133b, miR-206 and miR-103a-3p were used. The miRNAs expression levels were quantified using the 2^−ΔCt^ method, and miR-103a-3p as a relative control, as selected in our previous paper [14].

### 2.9. Immunofluorescent Staining

RH30 cells were fixed in 4% formaldehyde (POCH) in PBS, permeabilized in 0.1% TritonX-100 (Sigma-Aldrich) and blocked in 5% goat serum (ThermoFisher Scientific) together with 1% bovine serum albumin (BSA, Sigma-Aldrich) in PBS. Subsequently, the cells were incubated with mouse anti-PAX7 antibody (DSHB, Developmental Studies Hybridoma Bank, IA, USA) and then incubated with secondary goat anti-mouse antibodies conjugated with Alexa Fluor 488 (Life Technologies) and Hoechst 33342 (Life Terchnologies). The stained slides were mounted in Dako Fluorescence Mounting Medium (Dako, Denmark).

Microscopic images were collected using an Olympus BX51 or IX70 microscope (Olympus Corporation, Tokyo, Japan) and Olympus XC50 camera with cellSens Dimension software (both from Olympus). The images were processed using cellSens Dimension software.

### 2.10. In Vivo Experiments

Animal experiments were approved by the Local Ethics Committee in Krakow in Poland (no. 12/2018 with modifications 208A/2018 and 212/2018). In total, 5 × 10^6^ RH30 cells were injected subcutaneously into 6- to 8-week-old NOD-SCID mice. Each experimental group contained in total eight animals. The experiments were repeated twice, using a group containing four animals for each condition. Tumor size was evaluated using a caliper. Tumor volume was estimated using the formula V = D × d^2^ × 0.5 (where V is the tumor volume, D is the largest dimension, and d is the smallest dimension). After 23 days, the mice were euthanized, and their tumors were harvested. Following the evaluation of tumor weight, the tumor sections were fixed in formalin and stained with hematoxylin-eosin using Dako EnVision Detection Systems (Dako Polska Sp. z o.o., Poland) to visualize tumor morphology. After deparaffinization the tumor sections were stained immunohistochemically with anti-Ki67 primary mouse monoclonal antibody to evaluate tumor proliferation (clone MIB-1; 1: 75, DakoCytomation, Denmark, UK), and anti-CD31 antibody to visualize tumor vascularization (1:50, Abcam, ab28364), as described previously [13].

### 2.11. Analysis of RMS Tumor Samples from Patients

Human experiments were approved by the Local Bioethical Committee of the Collegium Medicum of the Jagiellonian University in Krakow, Poland (no. KBET/32/B/2014). The biopsies of eight RMS tumors were collected during routine surgery for the preparation of paraffin-embedded samples. Total RNA from paraffin-embedded tumor samples from patients was isolated using the RecoverAll™ Total Nucleic Acid Isolation Kit (Ambion). Subsequently, the analysis of the gene expression profile was performed as described above. 

### 2.12. Bioinformatic Analysis of Literature Data from RMS Cell Lines and Tumors

From literature data published by Gryder et al. 2017 in Supplemental Table S3 [32] the expression data in FPKM (fragments per kilo base per million mapped reads) was extracted for 25 cell lines and 90 tumor samples assigned to rhabdomyosarcoma type. Samples containing fusion oncogenes other than PAX3-FOXO1 were removed from the analysis. Then, expression levels in fusion-negative and fusion-positive tissues/cell lines were compared and presented as graphs of means ± SEM with statistical analysis.

### 2.13. Bioinformatic Analysis of miRNA Targets

Bioinformatic analysis of miRNA targets was performed with miRDB (http://mirdb.org/) [33] and TargetScanHuman 7.1 (http://www.targetscan.org/vert_71/) [34]. The online website tools were accessed on 7 July 2021.

### 2.14. Statistical Analysis

Unless stated otherwise, the results show the mean ± standard error of the mean (SEM) of at least three to four independent biological experiments, as stated in figure legends (n value). Statistical analysis was performed by one-way analysis of variance (ANOVA) with Tukey post-test for comparison of more than two groups or Student’s *t*-test and Mann–Whitney nonparametric test for comparison of two groups using GraphPad Prism software. Differences with a *p*-value less than 0.05 were considered statistically significant.

## 3. Results

### 3.1. RH30 Subclones of Different Origin Display Differences in Morphology, Proliferation Rate, and Migration

**Figure 1:**
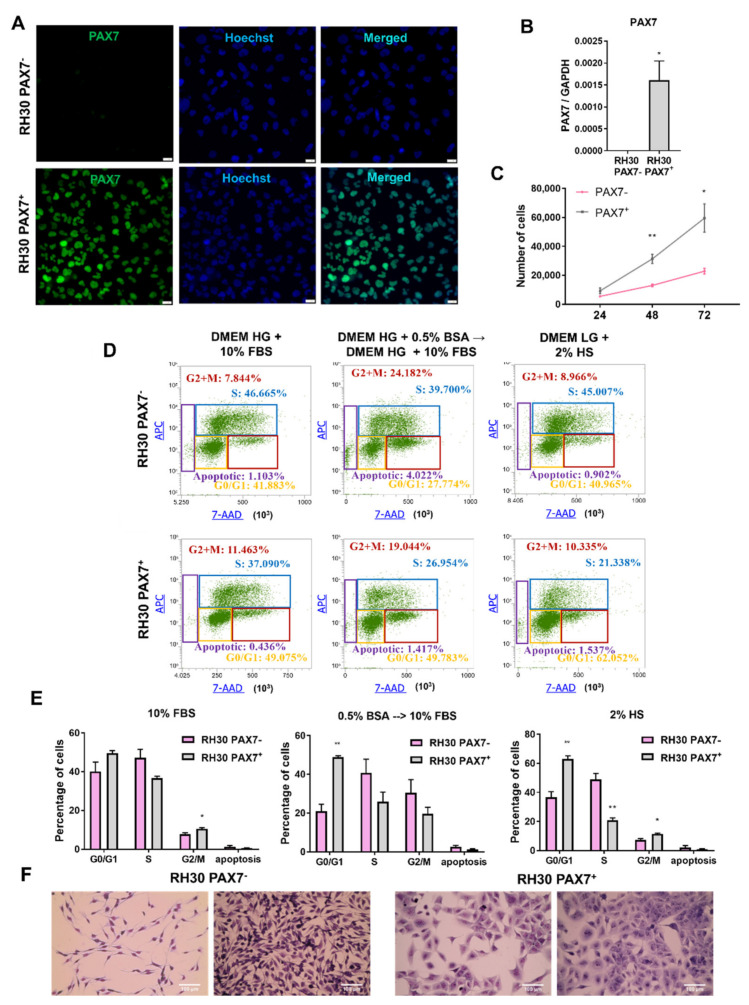
RH30 ARMS cells displayed clonal differences in PAX7 expression, morphology, and proliferation depending on their origin. (**A**) RH30 cells from ATCC are PAX7 negative, whereas RH30 cells donated by PJ Houghton were PAX7 positive. PAX7 protein was visualized with immunofluorescent staining and representative images are shown (green—PAX7, blue—nuclei, Hoechst). White scale bar represents 20 μm. (**B**) PAX7 mRNA expression levels evaluated with qPCR. Data were calculated with 2^−^^ΔCt^ method using GAPDH as a constitutive gene, *n* = 3. (**C**) PAX7^+^ positive cells proliferated faster in standard growth medium, as indicated by growth curve that shows the results of cell counting. The data on the graph show means ± SEM, *n* = 3. (**D**) RH30 subclones displayed differences in cell cycle and proliferation in three different conditions: (1) standard growth medium DMEM high glucose (HG) with 10% FBS for three days; (2) starvation medium consisting of DMEM with 0.5% BSA for two days and then DMEM with 10% FBS for one day; and (3) differentiating conditions in DMEM low glucose (LG) with 2% HS. Representative images of flow cytometry analysis are shown. The color of each gate corresponds to the color of the text indicating percentages (**E**) Differences in cell cycle between RH30 subclones are additionally shown in graphs summarizing three independent experiments. The data on graphs show means ± SEM. (**F**) RH30 subclones differed in morphology and size of the cells. Representative images of Wright’s staining are shown. White scale bar represents 100 μm. * *p* < 0.05, ** *p* < 0.01.

RH30 cells from two different origins: bought from ATCC or donated; were characterized for STR profile. Comparison with the data from the Expasy database showed that both RH30 cell lines displayed the correct STR profile (Table 1). Nevertheless, analysis of protein and mRNA expression profiles revealed that they turned out to be PAX7^−^ (ATCC) and PAX7^+^ (donated) cell line subclones (Figure 1A,B). Therefore, the subclones were further characterized to identify novel mechanisms responsible for RMS growth and progression. 

Firstly, differences in proliferation rate of RH30 subclones were detected. Counting the cells in standard conditions (DMEM HG medium with 10% FBS) revealed that PAX7^−^ cells proliferated slower than RH30 PAX7^+^ cells (Figure 1C). Subsequently, we characterized cell cycle and BrdU incorporation in three different conditions: standard growth medium (DMEM HG with 10% FBS), standard medium preceded by a two-day starvation culture (0.5% BSA) and differentiation medium (DMEM LG with 2% HS). RH30 PAX7^+^ cells displayed higher percentage of the cells in G2/M phase than PAX7^−^ cells, what suggests that they proliferate faster in standard growth medium. Analysis of the population of cells cultured in standard medium, but preceded by a two-day starvation culture showed higher percentage of PAX7^+^ cells in G0/G1 phase than PAX7^−^. Culturing under differentiating conditions in medium with 2% horse serum (HS) indicated also a higher percentage of PAX7^+^ cells in the G0/G1 phase and lower in S phase than PAX7^−^ cells (Figure 1D,E), what suggests that PAX7^+^ cells might be more sensitive to starving differentiating conditions that may induce cell cycle arrest. Nevertheless, other explanations cannot be excluded.

Importantly, strong morphological differences were observed in those two RH30 subclones. RH30 PAX7^+^ cells were bigger, more flattened and had higher granularity than RH30 PAX7^−^ cells (Figure 1F). Nevertheless, in both subclones, cells more or less resembling cells of the other subclone were observed (Figure 1F). Furthermore, in PAX7^+^ subclones heterogeneity of PAX7 protein levels was detected (Figure 1A).

**Figure 2:**
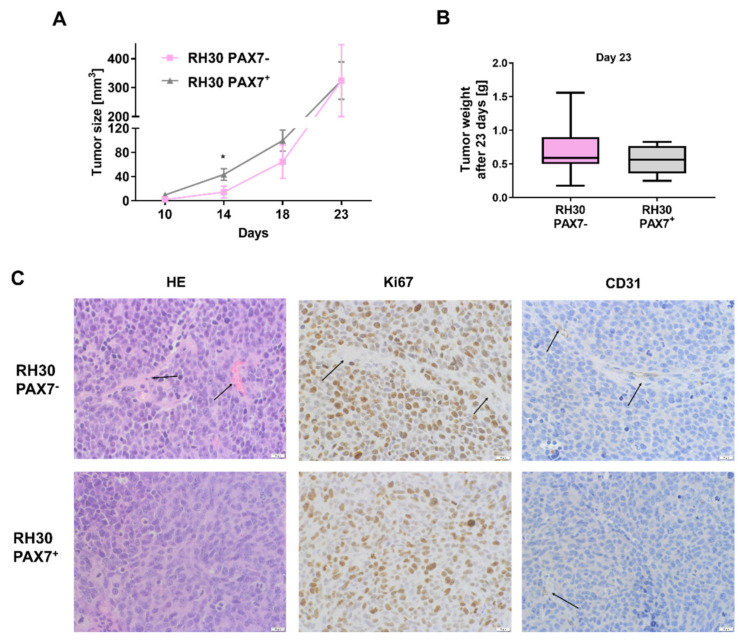
Xenotransplants of RH30 PAX7^−^ and PAX7^+^ subclones in immunodeficient NOD-SCID mice displayed slight differences in tumor morphology and tumor growth in several days after subcutaneous implantation. (**A**) RH30 PAX7^+^ subclone formed bigger tumors in NOD-SCID immunodeficient mice after subcutaneous implantation of the cells until 14 days. Tumor size was estimated with caliper, *n* = 8. (**B**) Tumors formed by RH30 subclones displayed similar weight 23 days after implantation; the results are presented as Whisker plot min to max, *n* = 8. (**C**) RH30 PAX7^+^ and PAX7^−^ subclones displayed slight differences in tumor morphology (hematoxylin-eosin staining), Ki67 level (brown color of dots) in fibrotic structures (arrows) and size of blood vessels (staining for CD31—brown color and arrows; blue color visualizes cellular morphology). Representative images of the staining are shown. White scale bar represents 20 μm. The data on graphs show means ± SEM. *p*-values are the results of Mann–Whitney nonparametric test. * *p* < 0.05.

To evaluate proliferation not only in vitro, tumor growth was also estimated in vivo after subcutaneous injection of the cells to immunodeficient NOD-SCID mice. The differences in tumor size were observed only until 14 days after implantation. RH30 PAX7^+^ cells more quickly started to form measurable tumors and possibly therefore they formed bigger tumors 14 days after implantation, but those differences disappeared in the next days (Figure 2A). Nevertheless, at the last day of the experiment, the growth and size of tumors in case of PAX7^−^ cells seemed to be more heterogeneous than in case of PAX7^+^ cells, due to the greater dispersion of the obtained results of measurements of the size and weight of tumors (Figure 2A,B). Those results may suggest that PAX7^−^ cells may be more sensitive to external conditions and tumor microenvironment, as previous studies suggested that tumor microenvironment may contribute in confounding ways to tumor progression and heterogeneity [13,35]. Additionally, tumors formed by RH30 subclones displayed only slight differences in tumor morphology, Ki67 level and CD31 positive blood vessels (Figure 2C).

**Figure 3:**
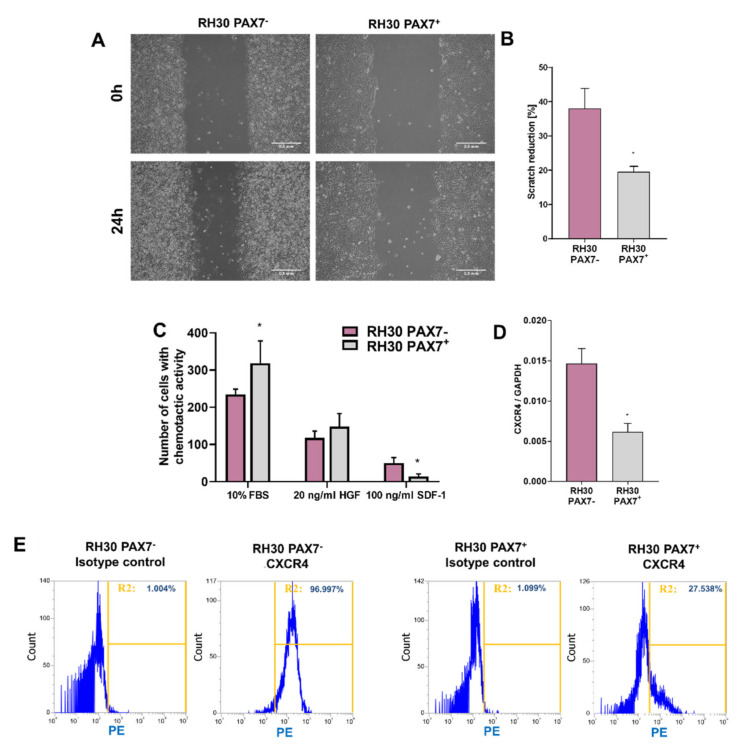
RH30 PAX7^−^ and PAX7^+^ subclones differed in migratory and chemotactic capabilities. (**A**) RH30 PAX7^−^ cells migrated faster in a scratch assay. Representative images of scratch assay are shown at time 0 and after 24 h from the same locations. Scale bar represents 500 μm. (**B**) RH30 PAX7^−^ cells displayed faster scratch reduction, *n* = 4. (**C**) RH30 PAX7^−^ cells had lower number of cells with chemotactic ability towards 10% FBS and higher towards 100 ng/mL SDF-1. No differences were shown between RH30 PAX7^−^ and RH30 PAX7^+^ in chemotactic ability towards 20 ng/mL HGF; *n* = 3. (**D**) RH30 PAX7^−^ cells displayed higher CXCR4 mRNA expression levels evaluated with qPCR. Data were calculated with 2^−^^ΔCt^ method using GAPDH as a constitutive gene, *n* = 3. (**E**) RH30 PAX7^−^ cells displayed higher level of CXCR4 receptor evaluated with flow cytometry. Representative images are shown. The data on graphs show means ± SEM. * *p* < 0.05.

Because for tumor progression not only proliferation, but also migratory capabilities of the cells are important, migration of the cells was estimated in a scratch assay. After 24 h, there were differences in closing a gap in a monolayer of the cells between PAX7^+^ and PAX7^−^ cells in starving conditions (Figure 3A,B). After testing the migratory capacity of RH30 PAX7^−^ and PAX7^+^ cells, their abilities to respond to specific chemoattractants were also tested. The number of RH30 PAX7^+^ cells migrating towards FBS gradient tended to be higher, and for SDF-1 lower compared to PAX7^−^ cells. The greater ability of PAX7^−^ cells to migrate in response to SDF-1 may result from a higher level of expression of CXCR4 receptor at mRNA (Figure 3D) and protein levels (Figure 3E), validated by qPCR and flow cytometry, respectively. No differences were observed in the number of HGF-responsive RH30 PAX7^−^ cells and PAX7^+^ cells (Figure 3C).

### 3.2. RH30 Subclones Display Differences in Expression Levels of Surface Markers and Myogenic Transcription Factors

To evaluate possible molecular pathways responsible for the observed differences between subclones, RH30 cells of different origin were also screened for expression of surface markers. Many differences were detected for multiple markers previously associated with tumor progression and adhesion of the cells (Table 2). Interesting example are three members of VLA family: CD49a, b, and c, that were highly expressed in RH30 PAX7^+^ cells. Moreover, ICAM family receptors, i.e., CD54 (ICAM-1) and CD102 (ICAM-2) were almost not expressed in RH30 PAX7^−^ cells. Higher expression of ICAMs and VLA integrins in RH30 PAX7^+^ cells may be responsible for tumor progression in vivo, as their levels were previously associated with tumor progression [14]. Among other markers upregulated in PAX7^+^ cells were: CD97, a widely expressed adhesion class G-protein-coupled receptor (aGPCR) that was previously found to be upregulated in RMS compared to skeletal muscles [36]; CD140B, known as PDGFRB important in RMS progression [37]; insulin receptor CD220 and insulin-like growth factor-1 receptor CD221, both associated with worse RMS survival [38] and activated leukocyte cell adhesion molecule (ALCAM), and CD166 associated with tumor invasiveness [39]. In contrast, among surface markers downregulated in PAX7^+^ cells were CD15 (SSEA1), which may serve as a marker of tumor-propagating cells [40] and sialophorin CD43 that mediates tumor cell-peritoneal adhesion [41].

**Figure 4:**
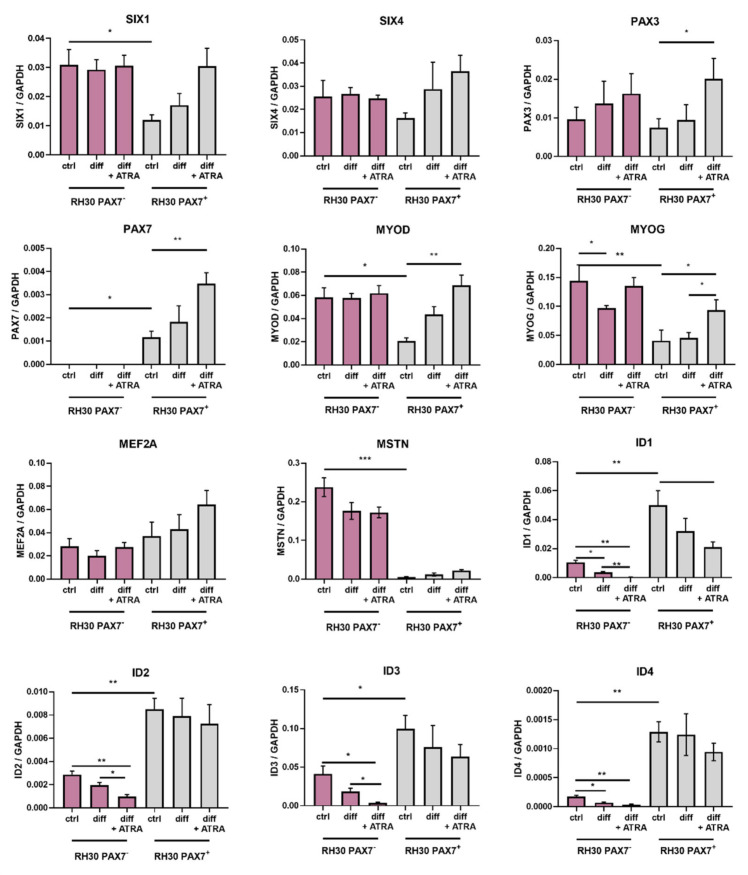
RH30 PAX7^−^ and PAX7^+^ subclones displayed differences in expression levels of transcription factors that regulate myogenic differentiation. RH30 subclones undifferentiated (ctrl) and differentiated in DMEM LG medium with 2% HS without or together with ATRA differed in expression levels of myogenic transcription factors and ID factors, *n* = 3. mRNA expression levels were evaluated with qPCR. Data were calculated with 2^−^^ΔCt^ method using GAPDH as a constitutive gene. The data on graphs show means ± SEM. * *p* < 0.05, ** *p* < 0.01, *** *p* < 0.001.

**Figure 5:**
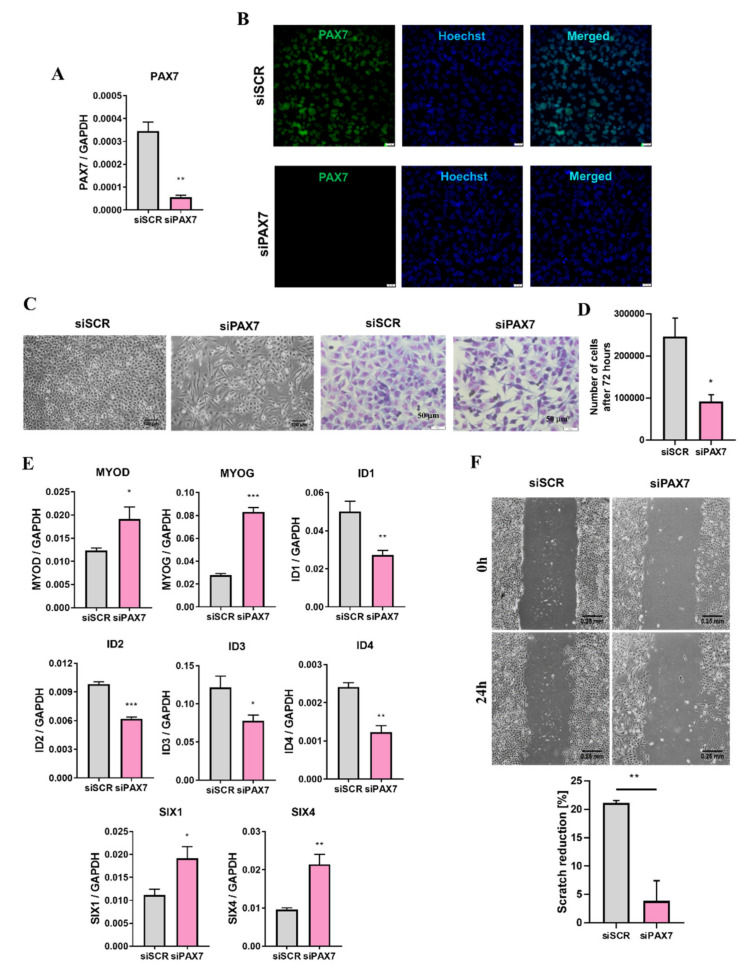
Silencing of PAX7 transcription factor in RH30 PAX7^+^ subclone enhanced myogenic differentiation, and inhibited proliferation and migration. (**A**) PAX7 mRNA level was downregulated after transfection of RH30 PAX7^+^ cells with siRNA (siPAX7) and control cells were modified with scrambled siRNA (siSCR), *n* = 3. (**B**) PAX7 protein silencing was visualized with immunofluorescent staining and representative images are shown (green—PAX7, blue—nuclei, Hoechst). White scale bar represents 20 μm. (**C**) PAX7 silencing in RH30 cells induced elongation of the cells and diminished their growth three days after transfection. Representative images of phase contrast (scale bar—100 μm) and Wright’s staining (scale bar—50 μm) are shown. (**D**) PAX7 silencing in RH30 cells diminished proliferation 96 h after transfection. The cells were seeded 24 h after transfection and then counted using Bürker chamber after the next 72 h, *n* = 3. (**E**) PAX7 silencing upregulated MYOD, MYOG, SIX1, and SIX4 levels, whereas downregulated ID1, ID2, ID3, and ID4 levels. mRNA expression levels were evaluated with qPCR. Data were calculated with 2^−^^ΔCt^ method using GAPDH or as constitutive genes, *n* = 3–4. (**F**) PAX7 silencing in RH30 cells diminished motility of the cells in a scratch assay. Representative images are shown (scale bar—250 μm) and graph with calculated scratch reduction, *n* = 3. The data on graphs show means ± SEM. * *p* < 0.05, ** *p* < 0.01, *** *p* < 0.001.

Besides surface markers, several transcription factors were also differentially expressed in RH30 subclones (Figure 4). As development of RMS results from abnormal differentiation of stem cells or early muscle progenitors, it was decided to test the ability of PAX7^−^ and PAX7^+^ cells to differentiate. For this purpose, the DMEM LG differentiation medium with 2% HS or an analogous medium with the addition of all-trans retinoic acid (ATRA) was used (Figure 5A). According to literature data, ATRA induces growth inhibition and at the same time stimulates myogenic differentiation [29,30,31]. Differentiation capabilities were compared in two differentiation media and standard growth medium. In those conditions, the expression of different factors that may regulate myogenic differentiation was investigated. RH30 PAX7^−^ cells expressed higher basal levels of SIX1, MYOD, MYOG, MSTN, and lower levels of ID1, ID2, ID3, and ID4 than RH30 PAX7^+^ cells (Figure 4), what suggests that they were more differentiated in standard growth conditions. Nevertheless, RH30 PAX7^+^ cells were more sensitive to induction of differentiation in vitro than PAX7^−^ cells, as the applied differentiation protocols induced expression of PAX3, PAX7, MYOD, and MYOG only in RH30 PAX7^+^ cells (Figure 4). PAX7^+^ cells start out as less differentiated and probably therefore the gene expression changes associated with the induction of differentiation were more profound.

### 3.3. PAX7 Transcription Factor Regulates Rhabdomyosarcoma Proliferation, Migration, and Differentiation

One of the crucial candidates differentially expressed between RH30 subclones is PAX7, as it is not present in RH30 ATCC cells (Figure 1A,B and Figure 4). To evaluate if the observed differences between two subclones are indeed PAX7 dependent and to emphasize its significance in ARMS, we silenced PAX7 expression with siRNA in RH30 PAX7^+^ cells. Control cells were transfected with scrambled siRNA. PAX7 silencing diminished PAX7 expression at both mRNA (Figure 5A) and protein levels (Figure 5B). PAX7 silencing induced morphological changes, as the PAX7 silenced cells were more elongated than the control cells (Figure 5C). PAX7 silenced cells displayed also diminished proliferation (Figure 5C,D). Inhibition of proliferation was accompanied by upregulation of MYOD and MYOG myogenic factors, inhibition of ID1, ID2, ID3, and ID4 factors, as well as upregulation of SIX1 and SIX4 (Figure 5E). Those results suggested that PAX7 silencing may induce myogenic differentiation of alveolar rhabdomyosarcoma. Interestingly, RH30 siPAX7 cells displayed slower migratory capabilities in a scratch assay (Figure 5F), whereas PAX7^−^ subclone migrated faster in that assay, but its migration towards FBS was indeed slower. Differences in migration capabilities in a scratch assay may be the effect of more complex dissimilarities in subclones besides the expression of PAX7. Those results suggest importance of further genomic and transcriptomic studies in the future.

**Figure 6:**
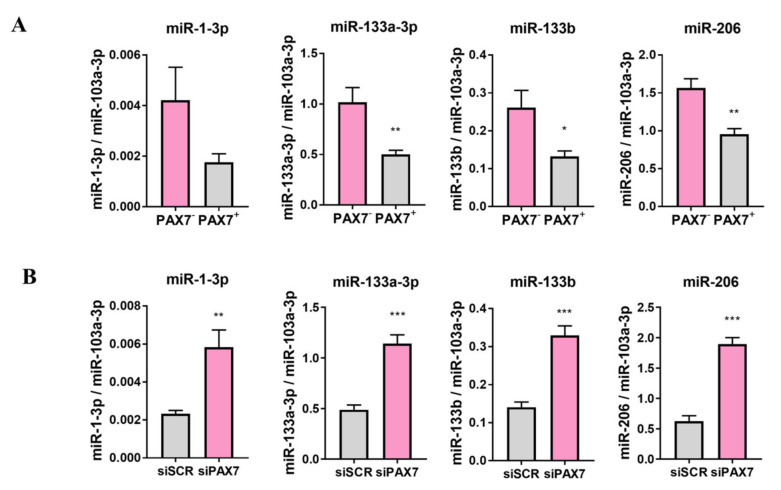
PAX7 transcription factor regulated expression levels of myogenic microRNAs in RH30 cells. (**A**) miR-1-3p, miR-133a-3p, miR-133b, and miR-206 expression levels were increased in RH30 PAX7^−^ subclone compared to RH30 PAX7^+^ subclone. (**B**) PAX7 silencing with siRNA in RH30 PAX7^+^ subclone upregulated miR-1-3p, miR-133a-3p, miR-133b, and miR-206 expression levels. miRNA expression levels were evaluated with qPCR, *n* = 3. Data were calculated with 2^−^^ΔCt^ method using miR-103a-3p as constitutive genes. The data on graphs show means ± SEM. * *p* < 0.05, ** *p* < 0.01, *** *p* < 0.001.

Moreover, PAX7 regulated not only myogenic regulatory factors, but also myogenic microRNAs. RH30 PAX7^−^ subclone cells displayed enhanced expression of miR-1-3p, miR-133a-3p, miR-133b, and miR-206 than PAX7^+^ cells (Figure 6A). Accordingly, PAX7 silencing with siRNA in RH30 PAX7^+^ cells induced expression of myogenic microRNAs, such as miR-1-3p, miR-133a-3p, miR-133b, and miR-206 (Figure 6B). Those results suggested that PAX7 may be a crucial regulator of rhabdomyosarcoma growth and an interesting candidate for further studies.

### 3.4. ERMS and ARMS Cell Lines Differentially Express Transcription Factors and microRNA That May Be Associated with Myogenic Differentiation

**Figure 7:**
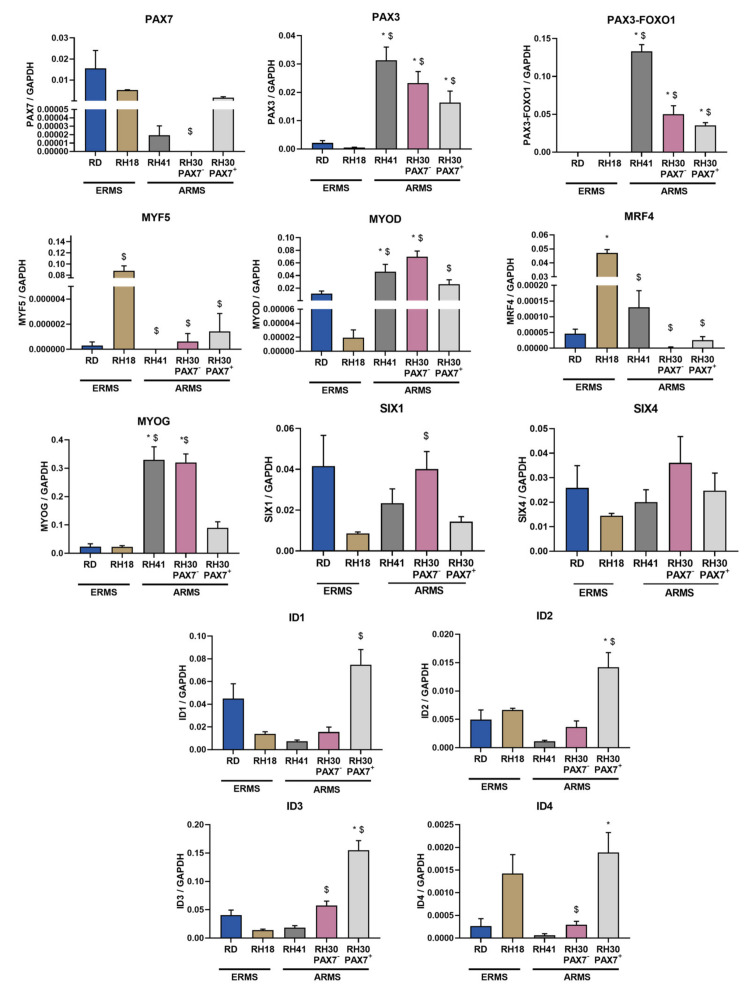
Transcription factors regulating myogenic differentiation were differentially expressed in ERMS and ARMS cells. mRNA expression levels were evaluated with qPCR. Data were calculated with 2^−^^ΔCt^ method using GAPDH as a constitutive gene. The data on graphs show means ± SEM, *n* = 3–4. Statistical analysis was performed with ANNOVA with Tukey posttest to analyze differences between ERMS and ARMS cell lines. Two types of differences were shown. * symbol indicates statistically significant differences compared to RD ERMS cell lines (*p* < 0.05), whereas $ symbol designates statistically significant differences compared to RH18 ERMS cell line (*p* < 0.05).

After performing a comparative analysis of the RH30 subclones, they were also compared with other RMS lines of the embryonic type (RD, RH18), as well as the alveolar type (RH41), to bring us closer to the identification of key genes for the progression of rhabdomyosarcoma (Figure 7). Importantly, PAX7 level was slightly elevated in ERMS cells, which further confirms its important role in RMS progression, that was described previously [20]. All our ARMS cell lines displayed expression of both PAX3 and PAX3-FOXO1, while in ERMS their lower expression or lack of expression was observed. ERMS cell lines displayed also lower expression of MYOG and MYOD. Other transcription factors were differentially expressed in different cell lines.

**Figure 8:**
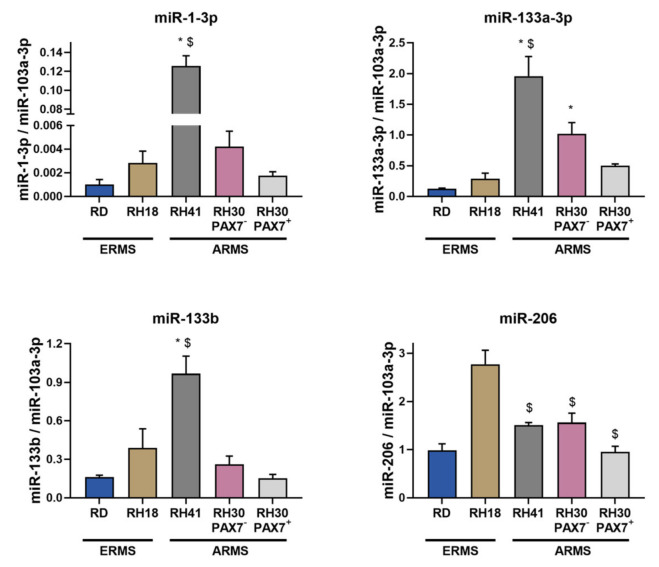
Myogenic microRNAs were differentially expressed in ERMS and ARMS cells. miR-1-3p, miR-133a-3p, miR-133b, and miR-206 expression levels were calculated with 2^−^^ΔCt^ method using miR-103a-3p as a constitutive gene. The data on graphs show means ± SEM, *n* = 3–4. Statistical analysis was performed with ANNOVA with Tukey posttest to analyze differences between ERMS and ARMS cell lines. Two types of differences were shown. * symbol indicates statistically significant differences compared to RD ERMS cell lines (*p* < 0.05), whereas $ symbol designates statistically significant differences compared to RH18 ERMS cell line (*p* < 0.05).

Subsequently, expression levels of myogenic microRNAs, including miR-1-3p, miR-133a-3p, miR-133b, and miR-206, were also evaluated in ERMS and ARMS cell lines (Figure 8). Differences were detected only in case of miR-133a-3p, as its levels were diminished in ERMS cell lines.

**Figure 9:**
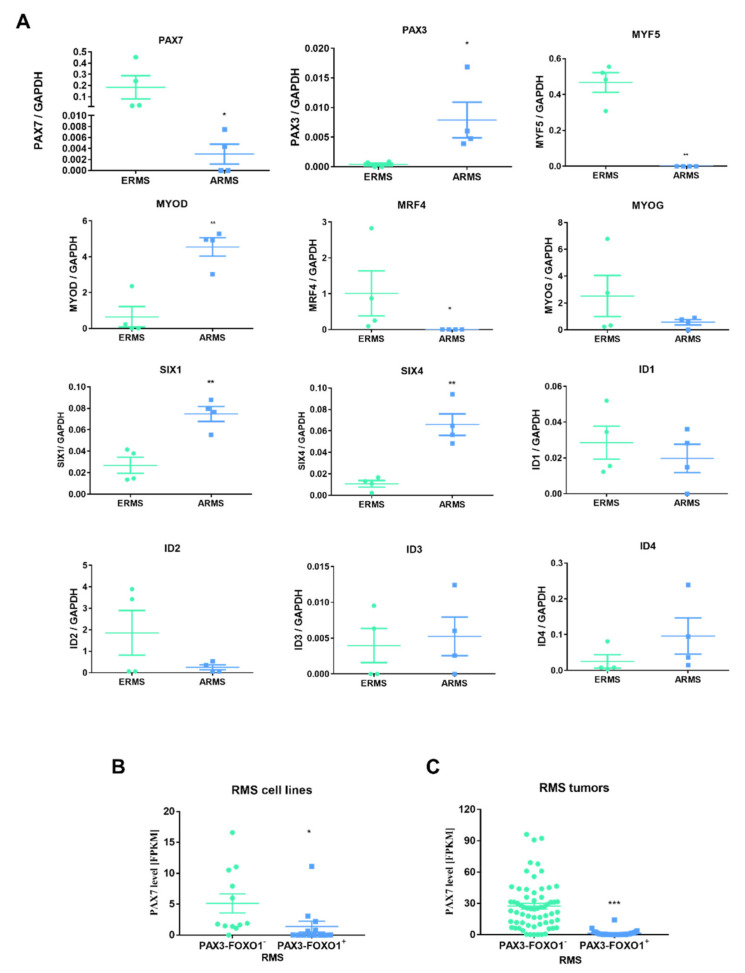
Transcription factors regulating myogenic differentiation were differentially expressed in ERMS and ARMS tumors in patients. (**A**) mRNA expression levels of different genes were evaluated with qPCR in four ERMS and four ARMS tumor samples from patients. Data were calculated with 2^−^^ΔCt^ method using GAPDH as a constitutive gene, *n* = 4. (**B**) PAX7 level in FPKM in 25 cell RMS lines positive and negative for PAX3-FOXO1 fusion extracted from the literature table results published by Gryder et al. 2017 [32] were reanalyzed and presented as dot plots. PAX7 level was diminished in ARMS cells positive for PAX3-FOXO1 fusion. (**C**) PAX7 level in FPKM in 90 RMS tumor samples positive and negative for PAX3-FOXO1 fusion extracted from the literature table results published by Gryder et al. 2017 [32] were reanalyzed and presented as dot plots. PAX7 level was diminished in RMS tumors positive for PAX3-FOXO1 fusion. The data on graphs is shown as dot plots with means ± SEM * *p* < 0.05, ** *p* < 0.01, *** *p* < 0.001.

Finally, to bring us closer to the identification of key genes for the progression of rhabdomyosarcoma, we evaluated expression levels of different genes regulating myogenic differentiation in four ERMS and four ARMS tumor samples from patients. That analysis showed lower PAX7, MYF5, and MRF4 levels in ARMS subtype than in ERMS, whereas higher levels of PAX3, MYOD, SIX1, and SIX4 (Figure 9A). Furthermore, we extracted also literature data of PAX7 expression levels in 25 RMS cell lines and 90 tumor samples positive or negative for PAX3-FOXO1 and presented them in dot plot graphs (Figure 9 B,C). Those results additionally confirmed heterogeneous expression of PAX7 in RMS and its higher levels in PAX3-FOXO1 negative than in PAX3-FOXO1 positive cell lines and tumors.

## 4. Discussion

We demonstrated that two subclones of RH30 cell line from different sources with the same STR profile, but with or without PAX7 expression, differ in multiple features, such as: morphology, proliferation, migration, chemotaxis, and cell growth in vivo. Those features of neoplastic cells may influence the malignancy of the tumor, its metastatic abilities, and the related effectiveness of therapy and favorable prognosis [23]. They are also important in the context of myogenic factors expression levels in rhabdomyosarcoma malignancy in patients [9]. In our studies, the observed differences in cellular parameters were also associated with dysregulation of transcription factors, microRNAs and surface markers.

The observed differences may arise from several reasons. The first is long-term culture, resulting in clonal selection in vitro and dominance of the original population by cells of a better adapted subline. Such events may take place both during cell culture in vitro and during tumor growth in vivo to favor population with evolutionarily more favorable features [21,22]. This instability has been also described for other cell lines previously, such as for example in malignant plasmacytoma cell lines [24] or the Ishikawa cell line [25], and our research additionally indicates it as an important feature. Previous results also demonstrated that the same cell lines validated with STR profiles may give different results depending on the clonal selection [24,25]. Importantly, RH30 line can be purchased from several cell line bases, including: DSMZ or ATCC and STR profiles of the RH30 line reported by the two organizations differ for some individual loci, according to Expasy Cellosaurus database [28]. This may suggest that the RH30 line was not homogeneous already at the stage of depositing in the databases. There are also single publications describing a point altered STR profiles [66], which additionally suggest a significant genetic instability of the described cell line. Moreover, according to some sources, the RH30 line is also identified as RMS13 line according to Expasy Cellosaurus database [28], while other literature references describe the RH30 and RMS13 as coming from the same patient, but completely separate lines [67]. According to a report published in Nature [68], more than 15% of the human cell lines used are not actually from the source quoted by the authors. Therefore, many journals require STR profile verification before publication.

Similarly to previous research [26,27], we used the tool of two RH30 subclones to identify novel molecular pathways influencing RMS progression. One of the crucial factors that was discovered by us as discriminating both subclones is PAX7, as one subclone is completely negative for this gene expression. It is also important to indicate that in the future, full genomic and transcriptomic characterization of two RH30 subclones should be performed, as it is indeed highly probable that different alterations besides PAX7 may be present in them.

Interestingly, previous studies on PAX3/7 in RMS were usually conducted in terms of the presence and importance of the PAX3/7-FOXO1 fusion genes [69]. Several studies focusing on the role of PAX7 in RMS lines negative for PAX3/7-FOXO1 fusion can be found [69], but the expression level and role of PAX7 in ARMS characterized by the PAX3-FOXO1 translocation are poorly understood. The role of PAX7 in ARMS did not catch the required attention probably because its levels are lower in fusion positive ARMS than fusion negative ERMS and its expression is heterogeneous. In this work, it was shown that PAX7 indeed plays an important role in a proliferation, migration, and in particular survival and differentiation of ARMS cells in cell line positive for the PAX3-FOXO1 translocation. Our results suggest that if PAX7 is present in PAX3-FOXO1 positive ARMS cells, downregulation of its level may force tumor to differentiate and inhibit its proliferation and progression. Interesting future direction is also validation in RMS patients whether diminished PAX7 levels in PAX3-FOXO1 positive ARMS tumors may be associated with better prognosis and survival of the patients. We validated PAX7 role using subclones and siRNA silencing, but in the future it is also worth to investigate the effects of its overexpression after transduction with viral vectors. The described trend of PAX7 effects in ARMS appear to be consistent with the current knowledge regarding the role of PAX7 in RMS and normal myogenesis. Other studies show that high levels of PAX7 in ERMS support cell migration capacity and invasiveness [70]. Moreover, increased expression of PAX7 in ERMS cells, as in the case of satellite cells [71], keeps them in a proliferative state and prevents the completion of the differentiation process [72]. Nevertheless, the role of PAX7 in RMS requires more detailed research in future. Up to date research showed that miR-206 mediated downregulation of PAX7 expression is essential for progressing ARMS cell differentiation [20]. Interestingly, our research demonstrated that PAX7 downregulation can also increase miR-206 levels, suggesting that there might be an interplay between those two factors. miR-206 may be also regulator of other factors important in myogenic differentiation. Bioinformatic analysis of potential binding sites with miRDB [33] and TargetScanHuman 7.1 [34] revealed miR-206 binding sites in PAX7 and PAX3. Furthermore, according to miRDB prediction data, miR-206 may also bind to ID4. What is also important, RH30 subclones displayed differential expression of miR-206. Similar effects were also detected for other myogenic microRNAs, such as miR-1-3p, miR-133a-3p, and miR-133b. Analysis of their targets and interactions in RH30 cells is an interesting direction for future studies.

Importantly, differentiation of RH30 subclones led to changes in the expression levels of not only PAX7, but also various transcription factors associated with myogenic differentiation. In the case of PAX7^+^ cells, during differentiation, there was a tendency to increase of the expression levels of SIX1, SIX4, PAX3, PAX7, MYOD, MYOG, MEF2A, and MSTN, whereas RH30 PAX7^−^ cells displayed higher basal levels of the selected factors that were not increasing further in differentiation media in vitro. Interestingly, in both subclones, ID factors expression levels tended to decrease during differentiation, which indicates also the role of ID factors in RMS that was not previously described in the literature. Furthermore, our results may suggest that differentiation in RMS lines that are at different stages of myogenesis may trigger different differentiation signaling pathways. The function of ID factors in inhibiting intramuscular differentiation at certain stages of normal myogenesis has been described previously [73] and both normal muscles [74] and stem cells [6] also express factors from this family. Our experiments with ERMS and ARMS lines and tumors showed that they express transcripts for all ID proteins, but their levels were different for each RMS line. The lowest total mRNA expression level for the ID factors was characteristic feature of RH41 line, which at the same time showed a relatively high level of expression of late myogenic markers in comparison to the other lines, i.e., in the case of MRF4 and MYOG. The ID1 and ID3 factors were characterized by the highest expression levels in the tested RMS lines. Coexpression of ID1 and ID3 factors, both in normal and neoplastic cells, is commonly observed, and their protein products usually perform the same functions [75]. Significantly higher levels of gene expression for ID1, ID3, and FOXO1 in the muscles of older men has been also described together with the correlation of the level of the ID1 factor with a decrease in muscle mass and strength [76], which may suggest a significant role of ID1/3 proteins in pathological conditions of muscles. Importantly, ID1 was identified as a factor expressed in undifferentiated and chemoresistant RMS cells [77].

Analysis of expression levels of other genes involved in myogenesis performed in different RMS lines also showed that MYOD and MYOG were expressed in all investigated cell lines. The level of MRF4 was practically undetectable in them, except for the RH18 line, which displayed a relatively high level. Nevertheless, previous results suggested that ARMS and ERMS lines, regardless of the subtype, showed MYOD and MRF4 expression, while the MYOG and MYF5 transcripts were detectable in all ARMS lines and most of the ERMS lines (five out of eight for MYOG and seven out of eight for MYF5) [74]. Most of the articles, however, show that MYF5 expression is at a higher level in ERMS and ARMS negative for PAX3/7-FOXO1 fusion [78].

Our research demonstrated that ERMS cells and tumors are characterized by a higher expression levels for the PAX7 gene, while ARMS cells for PAX3 and MYOD. Moreover, in all RMS lines higher MRF4 and MYF5 expression seemed to be associated with decreased MYOD and MYOG expression. These trends are in agreement with the previous studies of Tenente et al. 2017 [79]. Basing on the analysis of gene expression profiles from primary tumors samples, the group suggested the presence of two distinct genetic regulation systems dependent on MYOD or MYF5. The former was characterized by the simultaneous expression of MYF5, MRF4, and PAX7, while the second by the expression of MYOD, CDH15 (not tested in this study), and MYOG [79]. RH30 PAX7^+^ subclone compared to the PAX7^−^ subclone, showed higher levels of MYOD and MYOG expression, as well as seemingly lowered levels of PAX3. Therefore, the myogenic factor expression profile for this lineage appears to be slightly more shifted towards the embryonic type than that of RH30 PAX7^−^.^.^ This thesis is also supported by previous studies showing that the embryonic subtype cell lines are characterized by a lower level of CXCR4 compared to the alveolar subtype [10], which is consistent with the observed decreased level of CXCR4 expression for PAX7^+^ cells compared to PAX7^−^ cells.

The described RMS expression profiles may be due to the origin of these cells from different stages of intramuscular differentiation. Many studies describe RMS cells as derived from mesenchymal stem cells [80] or progenitors and differentiating myoblasts [81], which may be reflected by their expression profiles. For example, immunohistochemical analysis of tissue microarrays using anti-PAX7 antibodies showed a complete absence of PAX7 expression in 45% of ARMS cases, and only in 14% of ERMS cases, while PAX7 expression was focal in 83% of the analyzed ERMS cases [82]. This work indicates that ERMS is characterized by a higher level of PAX7 expression compared to ARMS and may suggest the origin of ERMS from satellite cells [83].

However, here the observed reverse pattern of expression for myogenic factors: PAX7, PAX3, MYOD, and MYOG, may be not related to a specific RMS subtype, but rather be a result of the presence or absence of the PAX3-FOXO1 fusion gene. We found that ERMS cells used in our research were negative for PAX3-FOXO1 fusion, in comparison to ARMS lines, positive for the genes fusion. These results are consistent with the literature data on the RMS lines [67]. There are studies showing that PAX3-FOXO1 induces the expression of the gene for PAX3, while it lowers the level of PAX7 expression [84]. In contrast, MYOG expression can be induced by PAX3-FOXO1 via a MYOD-independent pathway in ARMS cells [85].

Furthermore, our research showed also differences in migratory and chemotactic capabilities of RH30 subclones. Some of those differences may be associated with dysregulation of surface markers and receptors, such as CXCR4. CXCR4 receptor has been shown previously to regulate RMS cell trafficking, chemotaxis and adhesion in vitro [10]. Differences were also in expression of ICAM and VLA integrins, what may affect metastatic properties of those cells, as integrin adhesion to the ECM provides the traction required for tumor cell invasion [86] and may play a role in the post extravasation movement of RMS cells [14,87]. Among other markers differentially regulated in RH30 subclones were: CD97, a widely expressed adhesion class G-protein-coupled receptor (aGPCR) that was previously found to be upregulated in RMS compared to skeletal muscle [36]; CD140B, known as PDGFRB important in RMS progression [37]; insulin receptor CD220 and insulin-like growth factor-1 receptor CD221, both associated with worse RMS survival [38]; activated leukocyte cell adhesion molecule (ALCAM) CD166 associated with tumor invasiveness [39]; CD15 (SSEA1), which may serve as a marker of tumor-propagating cells [40] and sialophorin CD43 that mediates tumor cell-peritoneal adhesion [41].

## 5. Conclusions

To conclude, our results suggest that tumor cell lines with the same STR profile can produce subclones that differ in many features and may cause potential problems in reproducibility of the results between different research groups. Nevertheless, such subclones may be used as a model to identify novel crucial genes for tumor progression. RH30 rhabdomyosarcoma subclones indicated pivotal role of transcription factors associated with myogenic differentiation, such as PAX7 and ID proteins, in the development of RMS that should be investigated in future with focus on molecular mechanisms of their action. Interesting future direction is also validation in RMS patients whether diminished PAX7 levels in PAX3-FOXO1 positive ARMS tumors may be associated with better prognosis and survival of the patients.

## Figures and Tables

**Table 1:** STR profile of RH30 cells from two different sources: bought from ATCC or donated; in comparison to literature data from the Expasy database.

Loci	RH30 from ATCC	RH30 Donated	RH30 Data from the Expasy Database
D3S1358	15	15	15
vWA	17; 18	17; 18	17; 18
D16S539	12	12	12
D2S1338	17; 20	17; 20	17; 20
Amelogenin	X; Y	X	X (COG) or X; Y (ATCC)
D8S1179	12; 15	12; 15	12; 15
D21S11	29; 31.2	29; 31.2	29, 31.2
D18S51	15; 16	15; 16	15; 16
D19S433	14; 15.2	14; 15.2	14; 15.2
THO1	9; 9.3	9; 9.3	9; 9.3
FGA	22	22	22

**Table 2:** Differences in expression of surface markers in RH30 PAX7^−^ and PAX7^+^ clones. The results are shown as the percentage of cells labeled with a Lyoplate Screening Panel (flow cytometry).

Marker	Other Names	RH30 PAX7^−^ [%]	RH30 PAX7^+^ [%]	Significance in Tumor Biology
CD15	SSEA-1	68.97	3.61	Involved in tumor propagation; its expression increases proliferation in vitro and in vivo [40].
CD43	Sialophorin	67.99	3.05	Promotes cell growth by increasing cell survival, viability and colony formation; helps to evade FAS-mediated apoptosis [42]; ligand for ICAM-1—role in tumor cell-peritoneal adhesion [41].
CD49a	VLA-1; Integrin α1	36.25	96.24	Cell-surface receptor for collagens and laminins; involved in cell–cell adhesion and plays a role in inflammation and fibrosis; significantly upregulated in colorectal tumors thus promotes tumorigenicity and progressive capacity of this type of cancer [43]; associated with an invasive/metastatic phenotype; frequently overexpressed in malignancies such as melanoma, prostate, bladder, liver myeloma; a pre-malignant biomarker in pancreatic cancer [44].
CD49b	VLA-2; Integrin α2	4.33	85.29	It binds only to the β1 subunit (CD29). Involved in the metastatic activity and cell adhesion to an extracellular matrix (ECM) [45]; it participates in cell motility, angiogenesis and cell stemness; the role of integrin α2 in cancers is still not well understood and varies according to cancer type, i.e., angiogenesis effect [46].
CD49c	VL3A; Integrin α3	31.69	98.71	Involved in the increase of metastatic activity and promotion of adhesion to an extracellular matrix (ECM) [45]; may acts as pro-tumoral or tumoricidal factor depends on types of cancer, e.g., suppresses the metastasis of prostate cancer [47], whereas promotes migration and invasion of cervical cancer cells [48].
CD54	ICAM-1	4.25	96.04	Determines malignant potential of cancer via promotion of extraluminal migration of tumor cells [41]; key role during tumor progression and metastasis formation; involved in the activation of pro-inflammatory cascades, and the mediation of multiple signaling pathways that regulate metastasis, such as tumor cell adhesion and transmigration, immune escape, desmoplasia, and angiogenesis [49].
CD97	Adhesion G protein-coupled receptor E5	45.89	73.09	Elevated expression is associated with the dedifferentiation, aggressiveness, metastasis and invasion of tumor [50,51]; induced or upregulated, and/or biochemically modified in various malignancies. Involved in cell adhesion, migration, invasiveness, and regulation of intercellular junctions; affects tumor aggressiveness through stimulation of its ligand CD55 [52,53].
CD102	ICAM-2	0.01	23.72	Role in cellular adhesion; via interactions with extracellular matrix proteins plays a role in cell motility, i.e., might inhibit tumor cell motility and suppresses the metastatic potential [54]; ICAM-2 is underexpressed in human cancer tissues with p53 mutation; its low expression is associated with poor survival in patients with various cancers. Induction of ICAM-2 by p53 has a key role in inhibiting migration and invasion [55].
CD140b	PDGFRB	3.89	52.13	Promotes metastasis; tumor cells may acquire PDGFRB expression following epithelial-mesenchymal transition (EMT) during metastasis; it may contribute to form the aggressive phenotype of colorectal tumors with mesenchymal properties [56]; overexpression may lead to tumor cell growth and promote tumorigenesis; associated with certain malignant and non-malignant diseases characterised by extensive proliferation [57].
CD166	ALCAM	0.64	71.96	CD166 positive cells exhibit some CSCs-like (cancer stem cells) properties, such as sphere-forming ability, cell migration, adhesion, and high tumorigenic potential in vivo [58]; marker has been identified in many types of cancers; indicated as a biomarker for ovarian CSCs [58]; promotes cell migration, invasion, and metastasis in early-stage endometrial cancer [59]; plays a procarcinogenic role in liver cancer cells [60]; CD166 expression is positively correlated with the progression of breast cancer and melanoma [61,62].
CD220	Insulin receptor (IR)	20.08	59.36	Downregulation of the receptor inhibits cancer cell proliferation, angiogenesis and metastasis; IRs are highly expressed in malignant cells [63]; in many cancer cells, A isoform is more predominant form than isoform B; IRs have mitogenic effect promoting cancer growth [64].
CD221	IGF-1R	42.25	98.37	IGF-1R forms hybrid receptors with the isoform A of IR (IR-A), which are commonly overexpressed in human malignancies; hybrid receptors may be regard as potential targets of anti-cancer therapy; the inhibition of IGF-1R/IR-A activity may block cancer growth and metastatic spread [65].

## Data Availability

Data is contained within the article.

## References

[1] Yu P.Y., Guttridge D.C. (2018). Dysregulated myogenesis in rhabdomyosarcoma. Current Topics in Developmental Biology.

[2] Bentzinger C.F., Wang Y.X., Rudnicki M.A. (2012). Building muscle: Molecular regulation of myogenesis. Cold Spring Harb. Perspect. Biol..

[3] Relaix F., Demignon J., Laclef C., Pujol J., Santolini M., Niro C., Lagha M., Rocancourt D., Buckingham M., Maire P. (2013). Six Homeoproteins Directly Activate Myod Expression in the Gene Regulatory Networks That Control Early Myogenesis. PLoS Genet..

[4] Grifone R., Demignon J., Houbron C., Souil E., Niro C., Seller M.J., Hamard G., Maire P. (2005). Six1 and Six4 homeoproteins are required for Pax3 and Mrf expression during myogenesis in the mouse embryo. Development.

[5] Liu Y., Chu A., Chakroun I., Islam U., Blais A. (2010). Cooperation between myogenic regulatory factors and SIX family transcription factors is important for myoblast differentiation. Nucleic Acids Res..

[6] Ling F., Kang B., Sun X.H. (2014). Id proteins: Small molecules, mighty regulators. Current Topics in Developmental Biology.

[7] Langlands K., Yin X., Anand G., Prochownik E.V. (1997). Differential interactions of Id proteins with basic-helix-loop-helix transcription factors. J. Biol. Chem..

[8] Schaaf G.J., Ruijter J.M., Ruissen F., Zwijnenburg D.A., Waaijer R., Valentijn L.J., Benit-Deekman J., Kampen A.H.C., Baas F., Kool M. (2005). Full transcriptome analysis of rhabdomyosarcoma, normal and fetal skeletal muscle: Statistical comparison of multiple SAGE libraries. FASEB J..

[9] Skapek S.X., Ferrari A., Gupta A.A., Lupo P.J., Butler E., Shipley J., Barr F.G., Hawkins D.S. (2019). Rhabdomyosarcoma. Nat. Rev. Dis. Prim..

[10] Libura J., Drukala J., Majka M., Tomescu O., Navenot J.M., Kucia M., Marquez L., Peiper S.C., Barr F.G., Janowska-Wieczorek A. (2002). CXCR4-SDF-1 signaling is active in rhabdomyosarcoma cells and regulates locomotion, chemotaxis, and adhesion. Blood.

[11] Miekus K., Lukasiewicz E., Jarocha D., Sekula M., Drabik G., Majka M. (2013). The decreased metastatic potential of rhabdomyosarcoma cells obtained through MET receptor downregulation and the induction of differentiation. Cell Death Dis..

[12] Szewczyk B., Skrzypek K., Majka M. (2016). Targeting MET Receptor in Rhabdomyosarcoma: Rationale and Progress. Curr. Drug Targets.

[13] Skrzypek K., Kusienicka A., Szewczyk B., Adamus T., Lukasiewicz E., Miekus K., Majka M. (2015). Constitutive activation of MET signaling impairs myogenic differentiation of rhabdomyosarcoma and promotes its development and progression. Oncotarget.

[14] Skrzypek K., Kot M., Konieczny P., Nieszporek A., Kusienicka A., Lasota M., Bobela W., Jankowska U., Kędracka-Krok S., Majka M. (2020). SNAIL promotes metastatic behavior of rhabdomyosarcoma by increasing EZRIN and AKT expression and regulating microRNA networks. Cancers.

[15] Skrzypek K., Kusienicka A., Trzyna E., Szewczyk B., Ulman A., Konieczny P., Adamus T., Badyra B., Kortylewski M., Majka M. (2018). SNAIL is a key regulator of alveolar rhabdomyosarcoma tumor growth and differentiation through repression of MYF5 and MYOD function. Cell Death Dis..

[16] Ulman A., Skrzypek K., Konieczny P., Mussolino C., Cathomen T., Majka M. (2020). Genome Editing of the SNAI1 Gene in Rhabdomyosarcoma: A Novel Model for Studies of Its Role. Cells.

[17] Cieśla M., Dulak J., Józkowicz A. (2014). MicroRNAs and epigenetic mechanisms of rhabdomyosarcoma development. Int. J. Biochem. Cell Biol..

[18] Skrzypek K., Majka M. (2020). Interplay among SNAIL transcription factor, microRNAs, long non-coding RNAs, and circular RNAs in the regulation of tumor growth and metastasis. Cancers.

[19] Skrzypek K., Nieszporek A., Badyra B., Lasota M., Majka M. (2021). Enhancement of myogenic differentiation and inhibition of rhabdomyosarcoma progression by miR-28-3p and miR-193a-5p regulated by SNAIL. Mol. Ther. Nucleic Acids.

[20] Hanna J.A., Garcia M.R., Go J.C., Finkelstein D., Kodali K., Pagala V., Wang X., Peng J., Hatley M.E. (2016). PAX7 is a required target for microRNA-206-induced differentiation of fusion-negative rhabdomyosarcoma. Cell Death Dis..

[21] Gisselsson D., Lichtenzstejn D., Kachko P., Karlsson J., Manor E., Mai S. (2019). Clonal evolution through genetic bottlenecks and telomere attrition: Potential threats to in vitro data reproducibility. Genes Chromosom. Cancer.

[22] Porter S.N., Baker L.C., Mittelman D., Porteus M.H. (2014). Lentiviral and targeted cellular barcoding reveals ongoing clonal dynamics of cell lines in vitro and in vivo. Genome Biol..

[23] Hanahan D., Weinberg R.A., Francisco S. (2000). The Hallmarks of Cancer. Cell.

[24] Zhang X., Hou W., Epperly M.W., Rigatti L., Wang H., Franicola D., Sivanathan A., Greenberger J.S. (2016). Evolution of malignant plasmacytoma cell lines from K14E7 Fancd2-/- mouse long-term bone marrow cultures. Oncotarget.

[25] Kasai F., Hirayama N., Ozawa M., Iemura M., Kohara A. (2016). Changes of heterogeneous cell populations in the Ishikawa cell line during long-term culture: Proposal for an in vitro clonal evolution model of tumor cells. Genomics.

[26] Missiaglia E., Selfe J., Hamdi M., Williamson D., Schaaf G., Fang C., Koster J., Summersgill B., Messahel B., Versteeg R. (2009). Genomic imbalances in rhabdomyosarcoma cell lines affect expression of genes frequently altered in primary tumors: An approach to identify candidate genes involved in tumor development. Genes Chromosom. Cancer.

[27] Muff R., Rath P., Kumar R.M.R., Husmann K., Born W., Baudis M., Fuchs B. (2015). Genomic instability of osteosarcoma cell lines in culture: Impact on the prediction of metastasis relevant genes. PLoS ONE.

[28] Bairoch A. (2018). The cellosaurus, a cell-line knowledge resource. J. Biomol. Tech..

[29] Zhu G.H., Huang J., Bi Y., Su Y., Tang Y., He B.C., He Y., Luo J., Wang Y., Chen L. (2009). Activation of RXR and RAR signaling promotes myogenic differentiation of myoblastic C2C12 cells. Differentiation.

[30] Barlow J.W., Wiley J.C., Mous M., Narendran A., Gee M.F.W., Goldberg M., Sexsmith E., Malkin D. (2006). Differentiation of rhabdomyosarcoma cell lines using retinoic acid. Pediatr. Blood Cancer.

[31] Al-Tahan A., Sarkis O., Harajly M., Baghdadi O.K., Zibara K., Boulos F., Dighe D., Kregel S., Bazarbachi A., El-Sabban M. (2012). Retinoic acid fails to induce cell cycle arrest with myogenic differentiation in rhabdomyosarcoma. Pediatr. Blood Cancer.

[32] Gryder B.E., Yohe M.E., Chou H.C., Zhang X., Marques J., Wachtel M., Schaefer B., Sen N., Song Y., Gualtieri A. (2017). PAX3-FOXO1 establishes myogenic super enhancers and confers BET bromodomain vulnerability. Cancer Discov..

[33] Liu W., Wang X. (2019). Prediction of functional microRNA targets by integrative modeling of microRNA binding and target expression data. Genome Biol..

[34] Agarwal V., Bell G.W., Nam J.W., Bartel D.P. (2015). Predicting effective microRNA target sites in mammalian mRNAs. eLife.

[35] Runa F., Hamalian S., Meade K., Shisgal P., Gray P.C., Kelber J.A. (2017). Tumor microenvironment heterogeneity: Challenges and opportunities. Curr. Mol. Biol. Rep..

[36] Zyryanova T., Schneider R., Adams V., Sittig D., Kerner C., Gebhardt C., Ruffert H., Glasmacher S., Hepp P., Punkt K. (2014). Skeletal muscle expression of the adhesion-GPCR CD97: CD97 deletion induces an abnormal structure of the sarcoplasmatic reticulum but does not impair skeletal muscle function. PLoS ONE.

[37] Aslam M.I., Abraham J., Mansoor A., Druker B.J., Tyner J.W., Keller C. (2014). PDGFRβ reverses EphB4 signaling in alveolar rhabdomyosarcoma. Proc. Natl. Acad. Sci. USA.

[38] Shipley J., Martins A.S., Olmos D., Missiaglia E. (2011). Targeting the insulin-like growth factor pathway in rhabdomyosarcomas: Rationale and future perspectives. Sarcoma.

[39] Darvishi B., Boroumandieh S., Majidzadeh-A K., Salehi M., Jafari F., Farahmand L. (2020). The role of activated leukocyte cell adhesion molecule (ALCAM) in cancer progression, invasion, metastasis and recurrence: A novel cancer stem cell marker and tumor-specific prognostic marker. Exp. Mol. Pathol..

[40] Read T.A., Fogarty M.P., Markant S.L., McLendon R.E., Wei Z., Ellison D.W., Febbo P.G., Wechsler-Reya R.J. (2009). Identification of CD15 as a Marker for Tumor-Propagating Cells in a Mouse Model of Medulloblastoma. Cancer Cell.

[41] Ziprin P., Alkhamesi N.A., Ridgway P.F., Peck D.H., Darzi A.W. (2004). Tumour-expressed CD43 (sialophorin) mediates tumour-mesothelial cell adhesion. Biol. Chem..

[42] Kadaja-Saarepuu L., Laos S., Jääger K., Viil J., Balikova A., Lõoke M., Hansson G.C., Maimets T. (2008). CD43 promotes cell growth and helps to evade FAS-mediated apoptosis in non-hematopoietic cancer cells lacking the tumor suppressors p53 or ARF. Oncogene.

[43] Li H., Wang Y., Rong S.K., Li L., Chen T., Fan Y.Y., Wang Y.F., Yang C.R., Yang C., Cho W.C. (2020). Integrin α1 promotes tumorigenicity and progressive capacity of colorectal cancer. Int. J. Biol. Sci..

[44] Gharibi A., La Kim S., Molnar J., Brambilla D., Adamian Y., Hoover M., Hong J., Lin J., Wolfenden L., Kelber J.A. (2017). ITGA1 is a pre-malignant biomarker that promotes therapy resistance and metastatic potential in pancreatic cancer. Sci. Rep..

[45] Okazaki K., Nakayama Y., Shibao K., Hirata K., Nagata N., Itoh H. (1998). Enhancement of metastatic activity of colon cancer as influenced by expression of cell surface antigens. J. Surg. Res..

[46] Adorno-Cruz V., Liu H. (2019). Regulation and functions of integrin α2 in cell adhesion and disease. Genes Dis..

[47] Varzavand A., Hacker W., Ma D., Gibson-Corley K., Hawayek M., Tayh O.J., Brown J.A., Henry M.D., Stipp C.S. (2016). α3β1 integrin suppresses prostate cancer metastasis via regulation of the Hippo pathway. Cancer Res..

[48] Du Q., Wang W., Liu T., Shang C., Huang J., Liao Y., Qin S., Chen Y., Liu P., Liu J. (2020). High Expression of Integrin α3 Predicts Poor Prognosis and Promotes Tumor Metastasis and Angiogenesis by Activating the c-Src/Extracellular Signal-Regulated Protein Kinase/Focal Adhesion Kinase Signaling Pathway in Cervical Cancer. Front. Oncol..

[49] Benedicto A., Romayor I., Arteta B. (2017). Role of liver ICAM-1 in metastasis. Oncol. Lett..

[50] He Y., Xu L., Feng M., Wang W. (2019). Role of CD97 small isoform in human cervical carcinoma. Int. J. Exp. Pathol..

[51] Liu D., Trojanowicz B., Ye L., Li C., Zhang L., Li X., Li G., Zheng Y., Chen L. (2012). The invasion and metastasis promotion role of CD97 small isoform in gastric carcinoma. PLoS ONE.

[52] Tanase C., Gheorghisan-Galateanu A.A., Popescu I.D., Mihai S., Codrici E., Albulescu R., Hinescu M.E. (2020). Cd36 and cd97 in pancreatic cancer versus other malignancies. Int. J. Mol. Sci..

[53] Yin Y., Xu X., Tang J., Zhang W., Zhangyuan G., Ji J., Deng L., Lu S., Zhuo H., Sun B. (2018). CD97 Promotes Tumor Aggressiveness Through the Traditional G Protein–Coupled Receptor–Mediated Signaling in Hepatocellular Carcinoma. Hepatology.

[54] Yoon K.J., Miller A.L., Kreitzburg K.M. (2015). The role of ICAM-2 in neuroblastoma. Oncoscience.

[55] Sasaki Y., Tamura M., Takeda K., Ogi K., Nakagaki T., Koyama R., Idogawa M., Hiratsuka H., Tokino T. (2016). Identification and characterization of the intercellular adhesion molecule-2 gene as a novel p53 target. Oncotarget.

[56] Steller E.J.A., Raats D.A., Koster J., Rutten B., Govaert K.M., Emmink B.L., Snoeren N., van Hooff S.R., Holstege F.C.P., Maas C. (2013). PDGFRB promotes liver metastasis formation of mesenchymal-like colorectal tumor cells. Neoplasia.

[57] Heldin C.H. (2013). Targeting the PDGF signaling pathway in tumor treatment. Cell Commun. Signal..

[58] Kim D.K., Ham M.H., Lee S.Y., Shin M.J., Kim Y.E., Song P., Suh D.S., Kim J.H. (2020). CD166 promotes the cancer stem-like properties of primary epithelial ovarian cancer cells. BMB Rep..

[59] Devis L., Moiola C.P., Masia N., Martinez-Garcia E., Santacana M., Stirbat T.V., Brochard-Wyart F., García Á., Alameda F., Cabrera S. (2017). Activated leukocyte cell adhesion molecule (ALCAM) is a marker of recurrence and promotes cell migration, invasion, and metastasis in early-stage endometrioid endometrial cancer. J. Pathol..

[60] Yu W., Wang J., Ma L., Tang X., Qiao Y., Pan Q., Yu Y., Sun F. (2014). CD166 plays a pro-carcinogenic role in liver cancer cells via inhibition of FOXO proteins through AKT. Oncol. Rep..

[61] Jezierska A., Matysiak W., Motyl T. (2006). ALCAM/CD166 protects breast cancer cells against apoptosis and autophagy. Med. Sci. Monit..

[62] Swart G.W.M., Lunter P.C., Van Kilsdonk J.W.J., Van Kempen L.C.L.T. (2005). Activated leukocyte cell adhesion molecule (ALCAM/CD166): Signaling at the divide of melanoma cell clustering and cell migration?. Cancer Metastasis Rev..

[63] Zhang H., Fagan D.H., Zeng X., Freeman K.T., Sachdev D., Yee D. (2010). Inhibition of cancer cell proliferation and metastasis by insulin receptor downregulation. Oncogene.

[64] Vigneri R., Goldfine I.D., Frittitta L. (2016). Insulin, insulin receptors, and cancer. J. Endocrinol. Invest..

[65] Belfiore A. (2007). The Role of Insulin Receptor Isoforms and Hybrid Insulin/IGF-I Receptors in Human Cancer. Curr. Pharm. Des..

[66] Belyea B.C., Naini S., Bentley R.C., Linardic C.M. (2011). Inhibition of the notch-hey1 axis blocks embryonal rhabdomyosarcoma tumorigenesis. Clin. Cancer Res..

[67] Hinson A.R.P., Jones R., Lisa L.E., Belyea B.C., Barr F.G., Linardic C.M. (2013). Human rhabdomyosarcoma cell lines for rhabdomyosarcoma research: Utility and pitfalls. Front. Oncol..

[68] Masters J.R. (2012). Cell-line authentication: End the scandal of false cell lines. Nature.

[69] Seki M., Nishimura R., Yoshida K., Shimamura T., Shiraishi Y., Sato Y., Kato M., Chiba K., Tanaka H., Hoshino N. (2015). Integrated genetic and epigenetic analysis defines novel molecular subgroups in rhabdomyosarcoma. Nat. Commun..

[70] Chiappalupi S., Riuzzi F., Fulle S., Donato R., Sorci G. (2014). Defective RAGE activity in embryonal rhabdomyosarcoma cells results in high PAX7 levels that sustain migration and invasiveness. Carcinogenesis.

[71] Zammit P.S., Relaix F., Nagata Y., Ruiz A.P., Collins C.A., Partridge T.A., Beauchamp J.R. (2006). Pax7 and myogenic progression in skeletal muscle satellite cells. J. Cell Sci..

[72] Riuzzi F., Sorci G., Sagheddu R., Sidoni A., Alaggio R., Ninfo V., Donato R. (2014). RAGE signaling deficiency in rhabdomyosarcoma cells causes upregulation of PAX7 and uncontrolled proliferation. J. Cell Sci..

[73] Wang Y., Benezra R., Sassoon D.A. (1992). Id expression during mouse development: A role in morphogenesis. Dev. Dyn..

[74] Tonin P.N., Scrable H., Shimada H., Cavenee W.K. (1991). Muscle-specific Gene Expression in Rhabdomyosarcomas and Stages of Human Fetal Skeletal Muscle Development. Cancer Res..

[75] Lasorella A., Benezra R., Iavarone A. (2014). The ID proteins: Master regulators of cancer stem cells and tumour aggressiveness. Nat. Rev. Cancer.

[76] Buford T.W., Cooke M.B., Shelmadine B.D., Hudson G.M., Redd L.L., Willoughby D.S. (2011). Differential gene expression of FoxO1, ID1, and ID3 between young and older men and associations with muscle mass and function. Aging Clin. Exp. Res..

[77] Dawson L.E., D’Agostino L., Hakim A.A., Lackman R.D., Brown S.A., Sensenig R.B., Antonello Z.A., Kuzin I.I. (2020). Induction of Myogenic Differentiation Improves Chemosensitivity of Chemoresistant Cells in Soft-Tissue Sarcoma Cell Lines. Sarcoma.

[78] Zibat A., Missiaglia E., Rosenberger A., Pritchard-Jones K., Shipley J., Hahn H., Fulda S. (2010). Activation of the hedgehog pathway confers a poor prognosis in embryonal and fusion gene-negative alveolar rhabdomyosarcoma. Oncogene.

[79] Tenente I.M., Hayes M.N., Ignatius M.S., McCarthy K., Yohe M., Sindiri S., Gryder B., Oliveira M.L., Ramakrishnan A., Tang Q. (2017). Myogenic regulatory transcription factors regulate growth in rhabdomyosarcoma. eLife.

[80] Charytonowicz E., Cordon-Cardo C., Matushansky I., Ziman M. (2009). Alveolar rhabdomyosarcoma: Is the cell of origin a mesenchymal stem cell?. Cancer Lett..

[81] Rubin B.P., Nishijo K., Chen H.-I.H., Yi X., Schuetze D.P., Pal R., Prajapati S.I., Abraham J., Arenkiel B.R., Chen Q.-R. (2011). Evidence for an unanticipated relationship between undifferentiated pleomorphic sarcoma and embryonal rhabdomyosarcoma. Cancer Cell.

[82] Charville G.W., Varma S., Forgó E., Dumont S.N., Zambrano E., Trent J.C., Lazar A.J., Van De Rijn M. (2016). PAX7 expression in rhabdomyosarcoma, related soft tissue tumors, and small round blue cell neoplasms. Am. J. Surg. Pathol..

[83] Tiffin N., Williams R.D., Shipley J., Pritchard-Jones K. (2003). PAX7 expression in embryonal rhabdomyosarcoma suggests an origin in muscle satellite cells. Br. J. Cancer.

[84] Tomescu O., Xia S.J., Strezlecki D., Bennicelli J.L., Ginsberg J., Pawel B., Barr F.G. (2004). Inducible short-term and stable long-term cell culture systems reveal that the PAX3-FKHR fusion oncoprotein regulates CXCR4, PAX3, and PAX7 expression. Lab. Investig..

[85] Zhang L., Wang C. (2007). Identification of a new class of PAX3-FKHR target promoters: A role of the Pax3 paired box DNA binding domain. Oncogene.

[86] Desgrosellier J.S., Cheresh D.A. (2010). Integrins in cancer: Biological implications and therapeutic opportunities. Nat. Rev. Cancer.

[87] Hangan D., Uniyal S., Morris V.L., MacDonald I.C., von Ballestrem C., Chau T., Schmidt E.E., Chambers A.F., Groom A.C., Chan B.M. (1996). Integrin VLA-2 (alpha2beta1) function in postextravasation movement of human rhabdomyosarcoma RD cells in the liver. Cancer Res..

